# Two novel phages PSPa and APPa inhibit planktonic, sessile and persister populations of *Pseudomonas aeruginosa*, and mitigate its virulence in Zebrafish model

**DOI:** 10.1038/s41598-023-45313-x

**Published:** 2023-11-03

**Authors:** Chandrasekar Karthika, Nambiraman Malligarjunan, Ravi Jothi, Thirupathi Kasthuri, Rajaiah Alexpandi, Arumugam Veera Ravi, Shunmugiah Karutha Pandian, Shanmugaraj Gowrishankar

**Affiliations:** Department of Biotechnology, Science Campus, Alagappa University, Karaikudi, 630 003 Tamil Nadu India

**Keywords:** Biotechnology, Microbiology

## Abstract

The present study explores the avenue of phage therapy as an alternative antimicrobial therapeutic approach to counter multidrug-resistant (MDR) *Pseudomonas aeruginosa* infection. Our study investigated two novel virulent phages PSPa and APPa, specific to *P. aeruginosa,* in which in vitro evaluations were carried out to assess the therapeutic potential of phages. Both the identified phages exhibited host specificity by showing antagonistic activity of about 96.43% (27/28) and 92.85% (26/28) towards the 28 MDR clinical isolates of *P. aeruginosa*. The PSPa phage was found to have linear dsDNA with a sequence length of 66,368 bp and 92 ORFs, of which 32 were encoded for known functions of the phage life cycle and the remaining 60 were hypothetical functions. The APPa phage was found to have linear dsDNA with 59,591 bp of genome length and 79 ORFs, of which 15 were found to have known phage functions and the remaining 64 were found to be hypothetical proteins. Notably, the genome of both the phages lacks genes coding for tRNA, rRNA, and tmRNA. The phylogenetic analysis revealed that PSPa and APPa share > 95% sequence similarity with previously sequenced *Pseudomonas* viruses of their respective families. Further, the in vivo efficacy evaluation using the zebrafish model revealed that the treatment with PSPa and APPa has remarkably improved the survival rate of bacterial-infected zebrafish, reinforcing the anti-infective potential of the isolated phages PSPa and APPa against *P. aeruginosa* infection.

## Introduction

Globally, the incidence of bacterial infections is one of the most threatening public health emergencies in the last decades. As a consequence, the World Health Organization (WHO) has publicized a list of “priority pathogens”, to be considered critical and high-risk, which direly require urgent quest for new/novel antibiotics in clinics^1^. *Pseudomonas aeruginosa* is one of the dreadful multidrug-resistant (MDR) pathogens that topped the list^2^ owing to its increased rate of mortality and morbidity, especially in critically ill and immunocompromised individuals^3^. Adaptability to adverse environments and secretion of an array of virulence factors are attributed to this opportunistic pathogen to cause acute and/or chronic infections in patients suffering from cystic fibrosis, pulmonary disease, sepsis, traumas, and burn wounds^4^. Besides, the inherent characteristics of *P. aeruginosa* namely, rapid mutation and adaptation to gain resistance against antibiotics make the infection extremely difficult to treat in clinical settings^5^. An estimate by the Centers for Disease Control and Prevention (CDC) reports 32,600 cases, 2700 deaths, and a $767 million loss of cost on the account of treating *P. aeruginosa* infection in 2017^6^. A report by the Department of Biotechnology, Government of India signifies that 50% of *P. aeruginosa* isolates in India are resistant to broad-spectrum antibacterial agent—fluoroquinolones and third-generation beta-lactam antibiotic—cephalosporins^7^.

Another most alarming impediment to treating *Pseudomonas*-related biofilm infections is the persister cells. These cells eventually enter a metabolically dormant state during antibiotic/stress conditions, which thereby act refractory to antibiotics via activating efflux pumps and resume growth after antibiotic depletion. Hence, the traditional classes of antibiotics viz., beta-lactams, fluoroquinolones, and aminoglycosides remain ineffective against this dormant population^8^, while effective only towards metabolically active cells. Persister cells with their competency to limit antibiotics’ penetration and contact with other cells in a biofilm environment have been already correlated to the recalcitrance and relapse of chronic bacterial infections. Therefore, viable alternative treatment strategies targeting these transiently antibiotic-tolerant bacterial subpopulations are critically essential to effectively manage the clinical menace imposed by recurring/chronic biofilm infections, specifically cystic fibrosis^9^.

Of late, bacteriophages—nature’s predator of bacteria, with their long-term therapeutic values are being re-discovered by contemporary medicine in the midst of the global multidrug resistance era. Therefore, to circumvent this long-lasting issue of multidrug resistance in clinically important pathogens, especially *P. aeruginosa*, bacteriophage therapy holds greater promise and is perceived as an exciting and convenient alternative approach throughout the globe^10^.

Clinical practice of phage therapy has found its way a century ago since the discovery of phage in 1917 by Felix d’Herelle. To date, phage therapy remained active (either in combination with antibiotics or alone) in Eastern Europe, especially in countries such as Georgia and Poland through internationally renowned phage therapy centers^11^. However, in the rest of the world, physicians and researchers have been refocusing phage therapy as a compassionate last-resort therapeutic approach to balance the clinical needs of critically ill patients suffering recurrent bacterial infections owing to antibiotic failure. Despite the clinical advantages of phages as therapeutics [such as (1) ease of isolation; (2) proficiency in clearing off sessile cells; (3) self-replication at the infection site; (4) non-induction of side effects in treated individuals and; (5) adaptability to overcome resistance development in bacteria], randomized, controlled clinical trials using standard guidelines are being conducted globally to substantiate phage therapy as an alternative to antibiotics^12,13^.

Given the significance of phage therapy, the current study was deliberately focused on isolating and characterizing two virulent phages against MDR *P. aeruginosa* PAO1 (ATCC 15692). Additionally, efforts were also made to investigate the in vitro and in vivo therapeutic potencies of phages in inhibiting the growth of planktonic, sessile, and persister cells of *P. aeruginosa* PAO1 (ATCC 15692). The comprehensive efficacy analysis and in-depth characterization of two novel phages brought by this present investigation might contribute to their potential therapeutic utility in the treatment of MDR *P. aeruginosa* infections in the future.

## Materials and methods

### Bacterial strains used and their growth conditions

The reference strain of *P. aeruginosa* PAO1 (ATCC 15692) and twenty-eight MDR clinical isolates^14^ of *P. aeruginosa* (listed in Table 2) were obtained from Prof. S. Karutha Pandian, Alagappa University, and the other Gram-positive and Gram-negative microbial cultures (listed in Table 1) used in this study were maintained in Luria Bertani (LB) agar [containing enzymatic digest of casein (10.0 g/L), yeast extract (5.0 g/L), sodium chloride (10.0 g/L)] (HiMedia, India) as a host for phages. Prior to all experiments, the cultures were grown overnight in LB broth in an orbital shaker (Scigenics Biotech, India) at 130 rpm at 37 °C. From this, 100 μL of the bacterial suspension at logarithmic state (OD_600_ = 0.2; Spectramax M3, The USA) containing 1 × 10^8^ CFU/mL was used for all further assays namely double-layer agar (DLA), host range, adsorption, one-step growth curve, pH and temperature stability, planktonic cell lysis, mature biofilm disruption, and persister cell lysis assays. Pure bacterial cultures in LB were prepared as glycerol stock (25%) and stored at − 80 °C for long-term storage. All the experiments were performed using *P. aeruginosa* PAO1 ATCC 15692 unless otherwise mentioned.

**Table 1:** Spot test of phages PSPa and APPa on hosts of different genera.

S. No.	Bacterial strain	Activity (+/−)
1	*Pseudomonas aeruginosa* ATCC PA01	+
2	*Pseudomonas aeruginosa* PA14	+
3	*Pseudomonas otitidis* AATB4	−
4	*Klebsiella aerogenes *ATCC 35029	−
5	*Methicillin resistant Staphylococcus aureus* ATCC 33591	−
6	*Klebsiella pneumoniae* ATCC 700603	−
7	*Acinetobacter baumannii* ATCC 19606	−
8	*Enterococcus faecium* ATCC 51299	−
9	*Escherichia coli* MTCC	−
10	*Salmonella Typhi* MTCC 733	−
11	*Proteus mirabilis* ATCC 7002	−
12	*Vibrio alginolyticus* ATCC 17749	−
13	*Chromobacterium violaceum* ATCC 12472	−

### Phenotypic characterization

#### Isolation of phages capable of infecting *P. aeruginosa*

Two virulent phages (PSPa and APPa) were isolated and propagated from sewage [of Pallapatti, Dindigul (District)] and pond samples [of Ariyakudi, Sivaganga (District), Tamil Nadu] using the method suggested by De Melo et al.^15^ with slight modifications. A 0.5 mL test organism (PAO1) was mixed with 0.5 mL of 10× LB broth and added with 4 mL of wastewater sample before incubation at 37 °C for 24 h. Following incubation, the mixture was then centrifuged (ESCO, Singapore) at 7910 × *g* for 10 min at 37 °C. The supernatant was filtered through a 0.22 μm-pore-size hydrophilic polyethersulfone (PES) membrane filter (PALL Corporation, India) to remove bacterial debris, and the filtrate was collected. The presence of phages was determined using a spot test by making a spot using 10 µL of the filtrate over an LB plate of swabbed logarithmic bacterial culture and incubated at 37 °C (ORBITEK LEBT-D-1L, Sciengenics Biotech, India). After incubation, a clear zone was observed. These plaques containing phages were collected and added with sterilized sodium chloride-magnesium sulphate (SM) buffer (containing 0.1 M NaCl, 8 mM MgSO_4_, 50 mM Tris–HCl, 0.01% gelatin, and d. H_2_O maintained in pH 7.5) and kept in shaking at 4 °C for 4 h. Then, the samples were collected and treated with 0.5% chloroform, and centrifuged at 7910 × *g* for 10 min at 37 °C. Further, the supernatant was filtered through a hydrophilic PES membrane filter (PALL Corporation, India).

#### Purification of phages specific to *P. aeruginosa*

A single plaque from both phages was taken for subsequent purification by performing the DLA method as described by Kropinski et al.^16^ with required modifications. Purification of phages was done by continuously repeating the above-explained method until homogenous plaques were obtained. Following final purification, 0.5% chloroform-treated phage was used to infect *P. aeruginosa* cells in their early exponential growth phase. For 24 h, the infected cells were incubated at 130 rpm shaking (ORBITEK LEBT-D-1L, Sciengenics Biotech, India). The culture was centrifuged, and the supernatant was filtered through a sterile polyvinyl syringe filter with a pore size of 0.22-μm (PALL Corporation, India). Finally, purified phages were multiplied and stored at 4 °C.

#### Phage titration

The stored phage stock concentrates were used for the in vitro experiments and their titre was determined by the serial dilution method. Briefly, 100 µL of phage suspension is diluted with 900 µL of SM buffer to attain phages at different numbers. Following that, the DLA method was performed and the plates were incubated at 37 °C for 16 h. The plaque formation was observed, and the plaque-forming unit (PFU/mL) was calculated using the following equation:

$${\text{PFU}}/{\text{mL }}\; = \;{\text{ Number}}\;{\text{ of}}\;{\text{ plaques }}\; \times \;{\text{ Dilution }}\;{\text{factor}}/{\text{Volume}}\;{\text{ taken}}\; \, \left( {{\text{mL}}} \right)$$

By counting, the number of phage particles that infected the host bacterium (in a given colony forming unit (CFU), the multiplicity of infection (MOI) of the purified phage) was calculated by the following formula:

$${\text{MOI }} = {\text{ PFU}}/{\text{CFU}}.$$

#### Determination of host range and efficiency of plating (EOP)

To assess the lytic efficacy of isolated phages against 28 clinical MDR isolates of *P. aeruginosa* (Table 2), several Gram—positive and—negative standard bacterial pathogens (Table 2) were tested by spot assay method as explained in a previous study with slight modifications^17^. Briefly, 100 µL of exponentially developing clinical and reference strain of *P. aeruginosa* culture (1 × 10^8^ CFU/mL) was used to swab LB agar plates, and 10 µL of phage stock solution was spotted. Then plates were incubated for 16 h at 37 °C. The appearance of a zone of lysis at the spotted place was observed after incubation. In three independent tests, each of the phages was tested against each of the bacterial strains. The observed lysis was categorized into three as susceptible (+), less susceptible (0), and resistant (−).

**Table 2:**
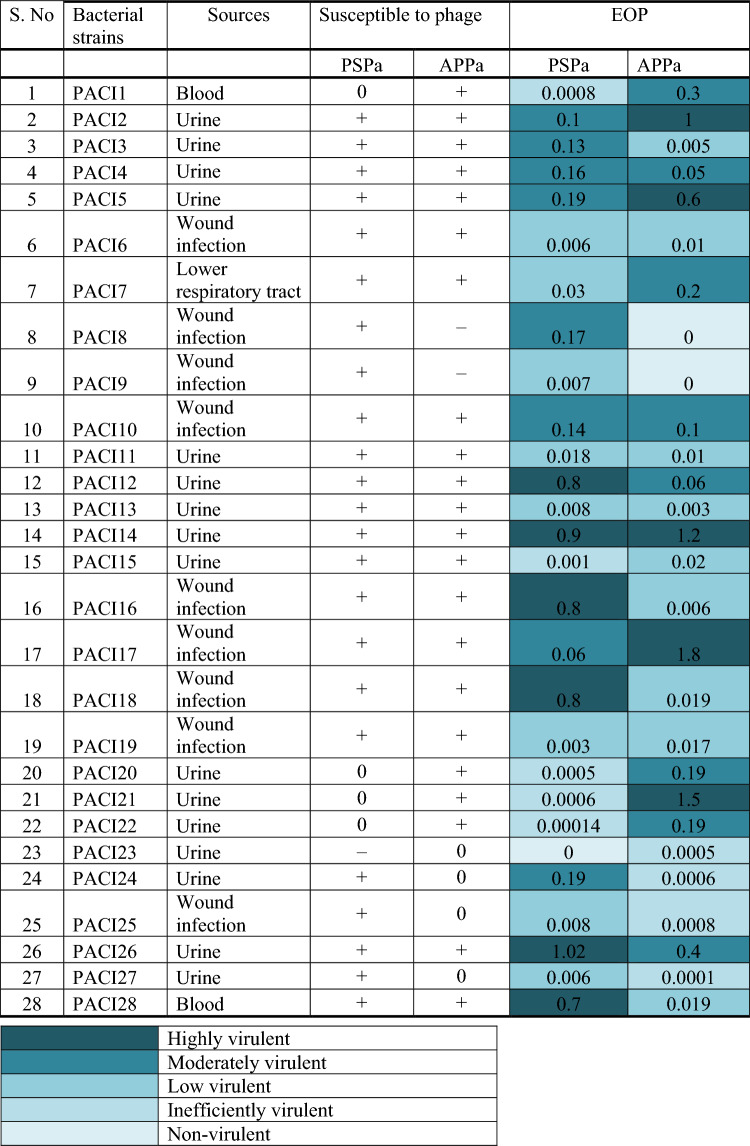
Results of spot test assay and efficiency of plating of different *P. aeruginosa* clinical isolates.

** + **(Susceptible), 0 (Less susceptible), − (Resistant).

Phages’ efficiency against different clinical isolates was determined using the DLA method.

EOP was calculated as the average PFU on clinical strain/average PFU on reference strain PAO1 (ATCC 15692).

By the obtained average EOP value, the phages were classified as highly virulent (≥ 0.5), moderately virulent (≥ 0.1 to < 0.5), low virulent (≥ 0.001 to < 0.1), inefficiently virulent (< 0.001) and non-virulent (if no plaques were detected).

#### Transmission electron microscopy

Transmission electron microscopy (TEM) was used to examine the morphological characteristics of the purified phages, as described previously by Kim et al.^18^ with required modifications. Both the phages after purification process were spotted onto carbon-coated 300-mesh copper grids (Sigma-Aldrich, The USA) followed by negative staining using 2% uranyl acetate. Finally, the micrographs were taken under a TEM (FEI TECNAI T12 Spirit, The Netherlands) at 60 kV.

#### Adsorption rate determination and one-step growth curve analysis

With some changes on a previously described method^19^, the adsorption rate and one-step growth kinetic studies were carried out for PSPa and APPa phages. Briefly, for the adsorption rate test, a 1 × 10^8^ CFU/mL host bacterial suspension along with phage suspension at a MOI of 0.001 was mixed and incubated at 37 °C. For every 2 min until 20 min, aliquots from the incubated sample (100 µL) were collected aseptically and centrifuged for 1 min at 9180 × *g*. Following that, the titre of the nonadsorbed phages were estimated in the collected supernatants using DLA method.

For one-step growth curve, host bacterial cells were grown in a 10 mL LB medium (1 × 10^8^ CFU/mL) and introduced to phage suspension at MOI of 0.001. The supernatants containing free phages were removed after 5 min of incubation by centrifugation at 9180 × *g* for 1 min, and the pellets were resuspended in a 50 mL fresh LB medium. For the next 80 min, 100 µL of culture samples were collected at 10 min intervals. The free bacteriophage count was determined by the DLA method at each time point. In this experiment, the latency period and burst period were identified using the following formula:

$${\text{Burst}}\;{\text{ size}}\, = \,{\text{the}}\;{\text{ phage}}\;{\text{ titres }}\;{\text{at}}\;{\text{ the }}\;{\text{plateau}}\;{\text{ phase}}/{\text{initial }}\;{\text{number}}\;{\text{ of }}\;{\text{infective}}\;{\text{ bacterial}}\;{\text{ cells}}{.}$$

#### Thermal and pH stability analysis

Effect of temperature and pH on the lytic efficacy of identified phages were assessed by following the previously described protocol^20,21^ with little alterations. Phage lysates (1.31 × 10^8^ PFU/mL) were incubated in a temperature-controlled water bath for 24 h at 4, 25, 37, 45, 50, 55, 65 and 70 °C. Following the incubation, the lysates were transferred to an ice-cold environment (− 20 °C) and proceeded with DLA method. The thermal stability of the two phages was tested in three parallel tests, and the phage titre was calculated using the DLA method. pH stability studies were performed using SM buffer and pH was adjusted using 1 N NaOH and 1 N HCl. For this experiment, 1.31 × 10^8^ PFU/mL of two phage suspensions (0.1 mL) were added to the SM buffer (0.9 mL) with a specific pH followed by incubation at 37 °C for 1 h. Then, the aliquots were removed for stability analysis and further tested for phage activity using the DLA method.

### Genomic characterization

#### Bacteriophage genomic DNA isolation and sequencing

Phage DNA was extracted from purified phage particles using the standard Phenol: Chloroform: Isoamyl alcohol (PCI) (25:24:1) method as described Sambrook and Russell^22^, with required modifications. Finally, the extracted phage DNA was precipitated with 70% ethanol, and DNA band was qualitatively visualized using 1% agarose gels. Whole-genome sequencing (WGS) of the extracted phage DNA was done [Macrogen, Inc. (Geumcheon-gu, Seoul, South Korea)]. The WGS was done using Illumina Hiseq. 2500 sequencer and de novo assembly were done using SPAdes and Platanus-allee software. Gapcloserv1.12. Artemis software (http://www.sanger.ac.uk/science/tools/artemis) was used to predict the putative open reading frames (ORFs) of the sequenced phage genome with a threshold of 30 amino acids as a minimum for the length of the protein. Functional annotation of the predicted ORFs was done to identify the putative functions of each ORF using BLAST tools at NCBI (E values of < 0.2) against non-redundant protein sequence databases. tRNAscan-SE (v1.23, http://lowelab.ucsc.edu/tRNAscan-SE) was used to identify Transfer RNAs and RNAmmer (v1.2, http://www.cbs.dtu.dk/services/RNAmmer/) was used to identify ribosome RNAs. To calculate the isoelectric points and molecular weights of the predicted proteins, the ExPASy Compute pI/Mw tool was used. The prokaryotic promoters' prediction was done using the BDGP prediction proGram (http://www.fruitfly.org/seq_tools/promoter.html). The sequence similarity of the whole viral nucleotides between closely related phages was determined using megablast at NCBI. The global alignment of putative amino acid sequences was performed using InterProscan v.5.30-69.0 and psiblast v2.4.0 for EggNOG DB. To assess the evolutionary development of the phages of interest, the phylogenetic analyses between the phages and their close relatives were done using ANI under pyani 0.2.10 of average_nucleotide_identity.py and ANIm way. Genomic comparisons between the sequenced phage genome with related phage genomes were done by Easyfig Software version 2.1.

### Assessment of therapeutic efficacy

#### Planktonic cell lysis assay

*P. aeruginosa.* phages PSPa and APPa were used in lysis experiments, by following previously described method with subtle changes^23^. The lytic activity of phages PSPa and APPa were evaluated using PAO1 ATCC (15692) in a liquid medium. First, 1% inoculum was inoculated in LB broth to obtain an overnight bacterial culture of *P. aeruginosa*. This pre-culture of bacteria was placed under shaker at 37 °C to obtain the logarithmic bacterial growth phase. The phages were combined with the cultivated host bacterium (0.2 at OD_600_) in fresh LB medium at MOI of 0.1, 1, and 10 in SM buffer. Spectrophotometer (Spectramax M3, The USA) at 600 nm was used to assess bacterial turbidity at 1-h intervals (1, 2, 3, 4, 5, 6, 7, 8, 9, 10, and 24 h) for 24 h with an experimental control devoid of phage.

CFU of the control and test samples was identified by spread plate method. Briefly, at each time point 1 mL of inoculum was collected from treated and untreated samples. The samples were serially diluted using sterile PBS and spread over LB agar plates. Then, incubated for 16 h at 37 °C and CFU/mL was calculated using the following formula:

$${\text{CFU}}/{\text{mL}}\; \, = \, \;{\text{Number}}\;{\text{ of }}\;{\text{colonies}}\; \, \times \, \;{\text{dilution }}\;{\text{factor}}/{\text{volume}}\;{\text{ taken}}\; \, \left( {{\text{mL}}} \right)$$

#### Mature biofilm assay

The biofilm inhibitory efficacy of the isolated phages was evaluated using the method previously described by Kim et al.^24^, with minor modifications. Briefly, 1% of the mid-log phase culture of PAO1 ATCC 15692 was inoculated into LB broth containing test tubes of two sets (one for CFU determination and PFU titration and the other for CV staining) and was allowed to form biofilm for 24 h 37 °C with shaking. Following incubation, the spent medium was discarded and the test tubes were washed carefully with sterile PBS to remove remaining unattached planktonic cells. Then, the adhered biofilm cells were replenished with fresh LB and treated with PSPa and APPa suspensions at 1.31 × 10^8^ PFU/mL individually, wherein, the untreated biofilm cells in LB served as control.

After treatment, spent medium containing planktonic cells and free phages was collected and centrifuged at 9180 × *g* for 5 min in the first set of test tubes at each time point, i.e., 0, 5, 10, 24 and 48 h, and 100 µL of the supernatant was used for DLA method to titre phages (PFU). Remaining adhered biofilm cells were washed twice with sterile PBS to remove remaining planktonic cells, and then resuspended in sterile PBS by vigorous vortexing until complete detachment was achieved, before being collected for CFU determination.

The second set of test tubes was used to quantify the biofilms formed using the CV staining method. The total biofilm biomass was determined by staining the biofilms for 15 min with 0.4% CV (HiMedia, India). Rinsing with sterile water removed the excess stain. The tubes were air-dried, and the CV stained cells were solubilised with 15% glacial acetic acid. At 570 nm, the OD of CV-bound biofilm cells were measured spectrophotometrically. The percentage of biofilm formation was calculated using the following formula:

$${\text{Formed}}\;{\text{ biofilms }}\left( \% \right)\, = \,\left[ {\left( {{\text{OD }}\;{\text{of}}\;{\text{ treated}}\;{\text{ sample}}} \right)/{\text{OD }}\;{\text{of}}\;{\text{ control}}\;{\text{ sample}}} \right]\, \times \,{1}00.$$

#### Alamar blue assay for biofilm cells

Alamar Blue assay was performed to quantitatively analyze the influence of phages on the cellular viability of *P. aeruginosa* biofilm at different time intervals following the protocol described earlier by Selvaraj et al.^25^. Briefly, PAO1 ATCC 15692 cells were allowed to form biofilm for 24 h. Following incubation, free-floating planktonic cells were removed and washed with sterile PBS. The remaining biofilm cells were suspended with sterile PBS and treated with (1.31 × 10^8^ PFU/mL) phages (PSPa and APPa, individually). After phage treatment, the metabolic viability of biofilm cells was assessed at predetermined time intervals (0, 5, 10, 24 and 48 h) by staining with Alamar blue. After staining with 2 µL of Alamar blue (10 mg/mL), the plates were kept in dark for 6 h at 37 °C. Finally, the fluorescent intensity was measured with excitation and emission at 530 nm and 590 nm, respectively using Multi-Mode Microplate Reader (SpectraMax® M3; The USA).

#### Microscopic analyses of *P. aeruginosa* biofilms

For the qualitative analysis of biofilm architecture, microscopy is one of the hallmark techniques, and thus the impact of phages on the sessile cells’ viability and 3D architecture was examined through three different microscopic techniques.

Biofilm assay was performed on glass slide pieces of 1 cm^2^ in 24-well MTP (Tarsons, India) containing 1 mL of LB and 1% of inoculum incubated for 24 h at 37 °C. After incubation, planktonic cells were washed twice with sterile PBS. Then, the well-bound biofilms were replenished with 1 mL of fresh sterile LB broth supplemented with or without (control) phages (1.31 × 10^8^ PFU/mL). The assay plate was statically incubated at 37 °C for 24 h. After incubation, the slides were stained (with appropriate dyes in accordance to the microscopy technique undergone) and observed using different microscopic analyses.

##### Light microscopic analysis

The light microscopic analysis was performed by following previously described protocol by Gowrishankar et al.^26^. The slides were washed gently with sterile PBS, air-dried and stained with 0.4% CV for 15 min. Again, washed with sterile PBS to remove the excess stain and let it to air-dry. Finally, the glass slides were examined under a light microscope (Nikon, Eclipse Ti-S; Tokyo, Japan) at 400 × magnification.

##### Fluorescence microscopic analysis

For the fluorescence microscopy, a live-dead stain assay was performed by following previously described protocol by Ravi et al.^27^. The slides were washed with sterile PBS, stained with 0.1% of acridine orange (AO) and ethidium bromide (EtBr) (1:1) (Sigma Aldrich) under dark conditions for 20 min. After washing with sterile PBS to remove the unbound stain, the slides were air-dried before examination under fluorescence microscope (Nikon, Eclipse Ts2R, Tokyo, Japan) at 400× magnification.

##### FE-SEM analysis

Field-emission scanning electron microscopic (FE-SEM; SUPRA 55VP, Carl Zeiss, Germany) analysis was performed by following the protocol described previously by Sushmitha et al.^28^. The biofilm grown on glass slides, after washing with sterile PBS, they were fixed using 2.5% glutaraldehyde for 4 h at 37 °C, and washed again with sterile PBS. Next, the slides were dehydrated with increasing concentrations (20, 40, 60, 80, and 100%) of ethanol. The dehydrated samples were gold sputtered before observation under FE-SEM.

#### Evaluation of PSPa and APPa lytic activity against ciprofloxacin-induced persister cells

Induction of persistence in PAO1 cells were done by following the protocol explained previously by Pan et al.^29^ with required modifications. Initially, 24 h formed PAO1 mature biofilm cells were treated with increasing concentrations (0.1–1024 µg) of ciprofloxacin (CIP) to determine the MIC. Following MIC determination, 24 h PAO1 biofilm cells were subjected to treatment with 100X MIC of CIP for 24 h at 37 °C.

After incubation, the spent medium was removed. The biofilm-residing persister cells were washed twice and resuspended with sterile 0.85% to remove residual CIP. The surviving persister cells of PAO1 were collected by disruption with vigorous shaking in the vortex. The isolated persister cells were used for phage treatment (PSPa and APPa, individually) at MOIs of 0.1, 1 and 10 for 6 h at 37 °C. The suspension with 100× CIP alone served as the negative control. The suspension devoid of 100× CIP and phages (PSPa and APPa) treatment was act as control. Following incubation, each of the samples was collected centrifuged and the pellet was washed to remove remaining phages. Then, pellet was suspended in sterile PBS, spread on LB plates and incubated for 24 h for visual assessment of growth and determination of CFU.

### In vivo characterization

#### Zebrafish maintenance

Adult wild-type zebrafish (> 6 months old) were procured from local aqua farm at Chennai, India, and were employed for in vivo studies to evaluate phages’ anti-infective efficacy. The obtained zebrafish were accustomed to laboratory condition by maintaining aquarium for a week by setting optimal sustenance parameters (28 ± 2 °C, pH 7.2 ± 0.5 and dissolved oxygen 7.07 ± 0.8 ppm), and fed with live *Artemia* twice a day. All the fish were maintained with 12 h photoperiod at 25 °C in the aquarium with aerated water. The in vivo experiment was approved by the Institutional Animals Ethics Committee (IAEC) of Alagappa University, Karaikudi (Ref. No.: 2007/GO/ReBi/S/18/CPCSEA dt 14.03.2018). The in vivo experiment with zebrafish was performed in accordance with ARRIVE guidelines and also following the guidelines of Committee for the Purpose of Control and Supervision of Experiments on Animals (CPCSEA), Government of India. The maintenance of fish was carried out by following OECD—Fish Embryo Acute Toxicity Test (FET) Guidelines^30^. All the in vivo experiments were performed in biological triplicates.

#### Toxicity assessment and in vivo anti-infective efficacy evaluation of phages against *P. aeruginosa* PAO1

The toxicity of phages on zebrafish was evaluated by calculating the survival rate. In this experiment, six fish were transferred into new tank containing phages PSPa and APPa at MOI 10. Then, the survival rate of the experimental fish was calculated after the exposure period of 96 h^31^.

For in vivo therapeutic efficacy assessment of PSPa and APPa, the healthy fish were infected with *P. aeruginosa* PAO1 (ATCC 15692) by introducing cell suspension of 1 × 10^8^ CFU/mL in freshwater for 12 h. Following that, six post-infected fish were transferred into new containers added with both the phages separately at MOI 10. PAO1-infected fish that were not exposed to phages acted as control. The uninfected fish served as naive control. Subsequently, the mortality in each group was observed up to 96 h and the percentage of survival rate was calculated by counting the number of dead animals^31^.

#### Histopathological examination

The experimental fish from control and phage treated conditions were subjected to histopathological analysis. The animals were anesthetized using Tricaine methanesulfonate (MS222) (164 mg/L; Sigma-Aldrich) and were fixed in 10% neutral-buffered formalin (NBF) solution for 24 h. The specimens were processed by dehydration, clearance in xylene and finally embedment in paraffin wax before being sectioned at 5 μm using a rotary microtome. The specimens were stained with hematoxylin and eosin. Finally, the stained sections were microscopically examined and imaged using a light microscope^32^.

### Statistical analysis

All the experiments were performed in biological triplicates with at least three technical triplicates. For statistical analysis unpaired t-test and ANOVA were used. GraphPad Prism v.7.04 has been used to analyse the data. *P* value of < 0.05 = *, *P* < 0.01 = **, *P* < 0.001 = *** and *P* < 0.0001 = ****. All the statistical data were converted to graph using GraphPad Prism v.7.04.

## Results

### Phenotypic characterization

#### Isolation and titration of phages

**Figure 1:**
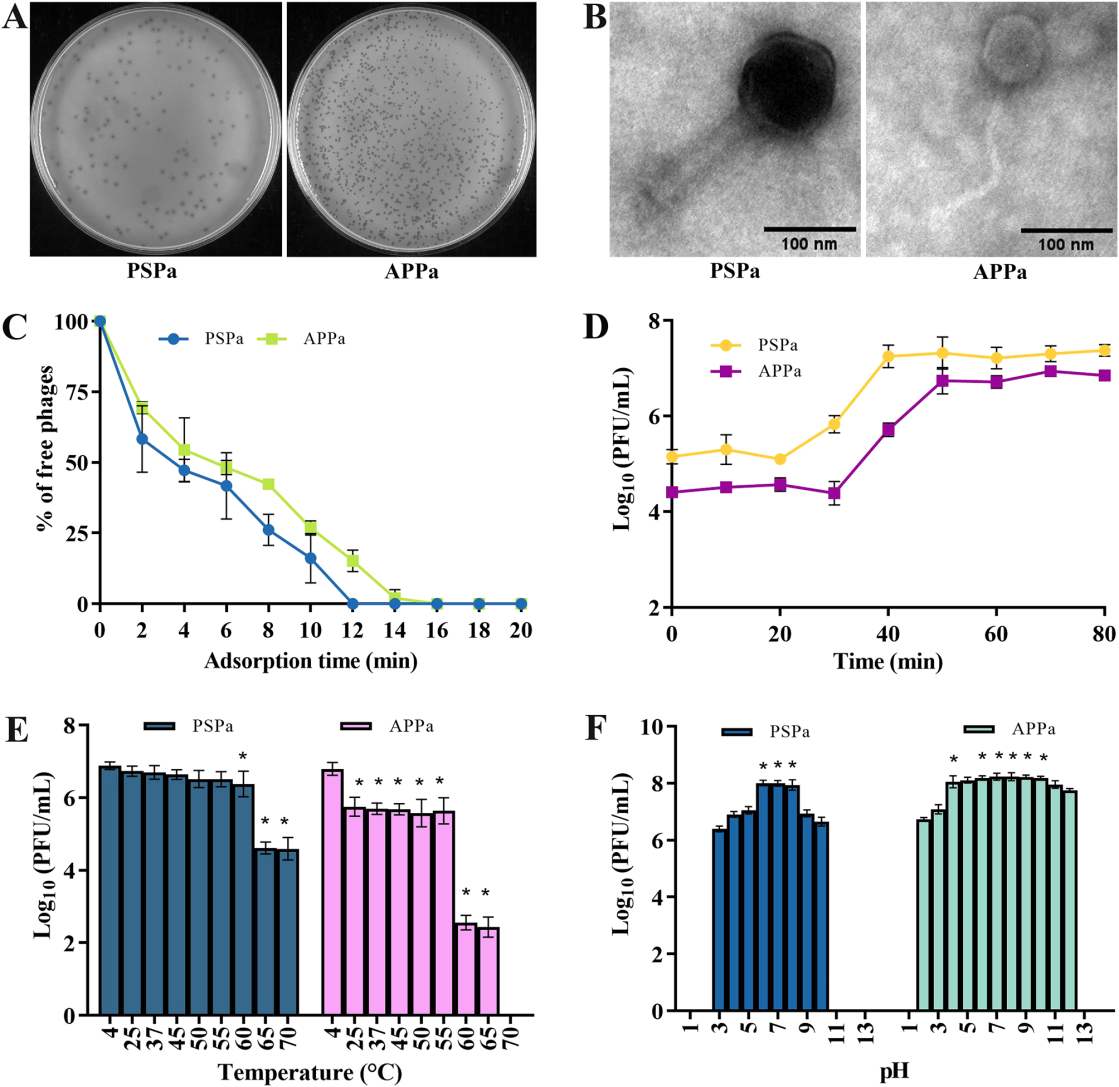
Phenotypic characterization of the two phages. (**A**) Soft agar overlay assay results showing uniform plaques of PSPa (Left) and APPa (Right) phages infecting *P. aeruginosa* strain PAO1 (ATCC 15692); (**B**) TEM micrographs of PSPa (Left) and APPa (Right) phages showing morphological structures with scale bar of 100 nm; (**C**) adsorption rate of PSPa and APPa; (**D**) one-step growth curves of PSPa and APPa; (**E**) thermal stability of phages PSPa and APPa observed at varying temperatures and; (**F**) pH stability of PSPa and APPa observed in varying pH at 37 °C. The *, **, *** and **** denotes the significant values *P* < 0.05, *P* < 0.01,* P* < 0.001 and *P* < 0.0001, respectively. All the data represent the mean standard deviation from triplicate experiments.

Phages, despite being ubiquitous in nature, sewage water bodies are an incredible source for screening of specific phages against pathogens. Primarily, two potent virulent phages, designated as PSPa and APPa that act against MDR *P. aeruginosa* PAO1 (ATCC 15692) were isolated from sewage and pond samples collected from Pallapatti (Dindigul District) and Ariyakudi (Sivaganga District) of Tamil Nadu, India. In spot test, a clear zone was observed over a bacterial lawn due to the lytic activity of PSPa and APPa. Two phages formed small but visible plaques of identical morphology on DLA **(**Fig. 1A: left—PSPa**,** right—APPa**)**. Bacteriophage multiplication was carried out by repeating the plaque purification method, and a stock of 1.31 × 10^8^ PFU/mL was used for further analysis.

#### Determination of host range and EOP against clinical isolates of *P. aeruginosa*

The host range of phages against bacterial hosts of different genera and different species (including clinical strains) of *Pseudomonas* were tested. The test results (Table 1) ascertained the specificity towards *P. aeruginosa* (see Supplemental Fig. 1 in the Supplemental material).

As shown in the Table 2, the results of spot assay test to assess the infectivity of isolated phages against *P. aeruginosa* (PAO1 (ATCC 15692) and 28 clinical isolates) suggest that the phages have highly virulent spectrum against the species PAO1 (ATCC 15692) and 28 clinical isolates (strains). In addition, the PSPa and APPa lysed 96.43% (27/28) and 92.85% (26/28) of *P. aeruginosa* clinical strains, respectively (Supplemental Figs. 2 and 3).

Phages PSPa and APPa were able to infect 27 and 26 out of 28 MDR *P. aeruginosa* clinical strains. Both the phages exhibited a range of efficiency towards 28 *P. aeruginosa* MDR clinical strains (Table 2). For PSPa, 21.42% of efficient activity was found at EOP of ≥ 0.5 and 57% of efficient activity in EOP of ≥ 0.001 to < 0.1. APPa exhibited 17.85% of efficiency in EOP of ≥ 0.5 and 32.14% of efficiency in EOP of ≥ 0.001 to < 0.1.

#### Nomenclature and morphological characterization of two phages

The Transmission Electron Microscopy (TEM) was employed to identify phages based on their morphological characteristics, as it is a standard technique for the classification of viruses. International Committee on Taxonomy of Viruses (ICTV) guidelines was followed for classifying both PSPa and APPa. The obtained results revealed both phages to be the members of the class *Caudoviricetes* which involves all tailed bacterial and archaeal viruses with icosahedral capsids and a double-stranded DNA genome^33^. The transmission electron micrograph of PSPa portrayed the typical morphological characteristic features i.e., isometric head having contractile tail in the extended state of *Straboviridae* family (Fig. 1B (left)). Whereas, APPa phage was found to be belonging to the family *Autographiviridae*, as the electron micrograph displayed an isometric head and an extremely long and flexible tail (as previously explained in ICTV guidelines^34^ before abolishment of the order *Caudovirales*) (Fig. 1B (right)). Phage PSPa has a capsid size of width (105 ± 2 nm), length (112 ± 1.5 nm) in diameter and a tail length of (161 ± 2 nm) and (24 ± 2 nm) wide, while Phage APPa has a capsid size of width (67 ± 1.7 nm), length (75 ± 1.9 nm) in diameter and a tail length of (179 ± 4 nm) and (9 ± 0.8 nm) wide.

#### Phage adsorption and growth kinetics

A one-step growth curve and adsorption period experiment were conducted to elucidate the phage's infection dynamics and its ability to infect and multiply within the host bacterial culture. The adsorption kinetics of PSPa and APPa phages were investigated, revealing a comparable adsorption rate, with both phages achieving more than 99% adsorption onto host cells within 12 min for PSPa and 16 min for APPa (as illustrated in Fig. 1C). Following adsorption, the latent period was assessed (as depicted in Fig. 1D), showing that PSPa and APPa phages exhibited latent periods ranging from 20 to 30 min, reaching a plateau at approximately 40–50 min, respectively. Their estimated burst sizes were 165 and 278 phage particles/infected cell, respectively.

#### Phage stability and viability in varied temperatures and pH

When the temperature (Fig. 1E) was raised to 65 °C, the infectivity of PSPa decreased and the infectivity remained constant at 70 °C. In contrast, phage APPa’s infectivity dropped dramatically at 60 °C and was completely lost at 70 °C (*P* < 0.05).

In pH stability (Fig. 1F), when exposed for 2 h to a pH range of 3–11, PSPa phage infectivity towards PAO1 was unaffected. The infectivity of PSPa was very stable in the pH range of 6–8 and moderately stable in the pH ranges of 3–5 and 9–10. In case of APPa phage, the infectivity remained stable in the pH range of 4–12 and slightly stable in the pH range of 2–3 (*P* < 0.05).

### Genomic characterization

#### Bacteriophage genomic DNA isolation and sequencing

**Figure 2:**
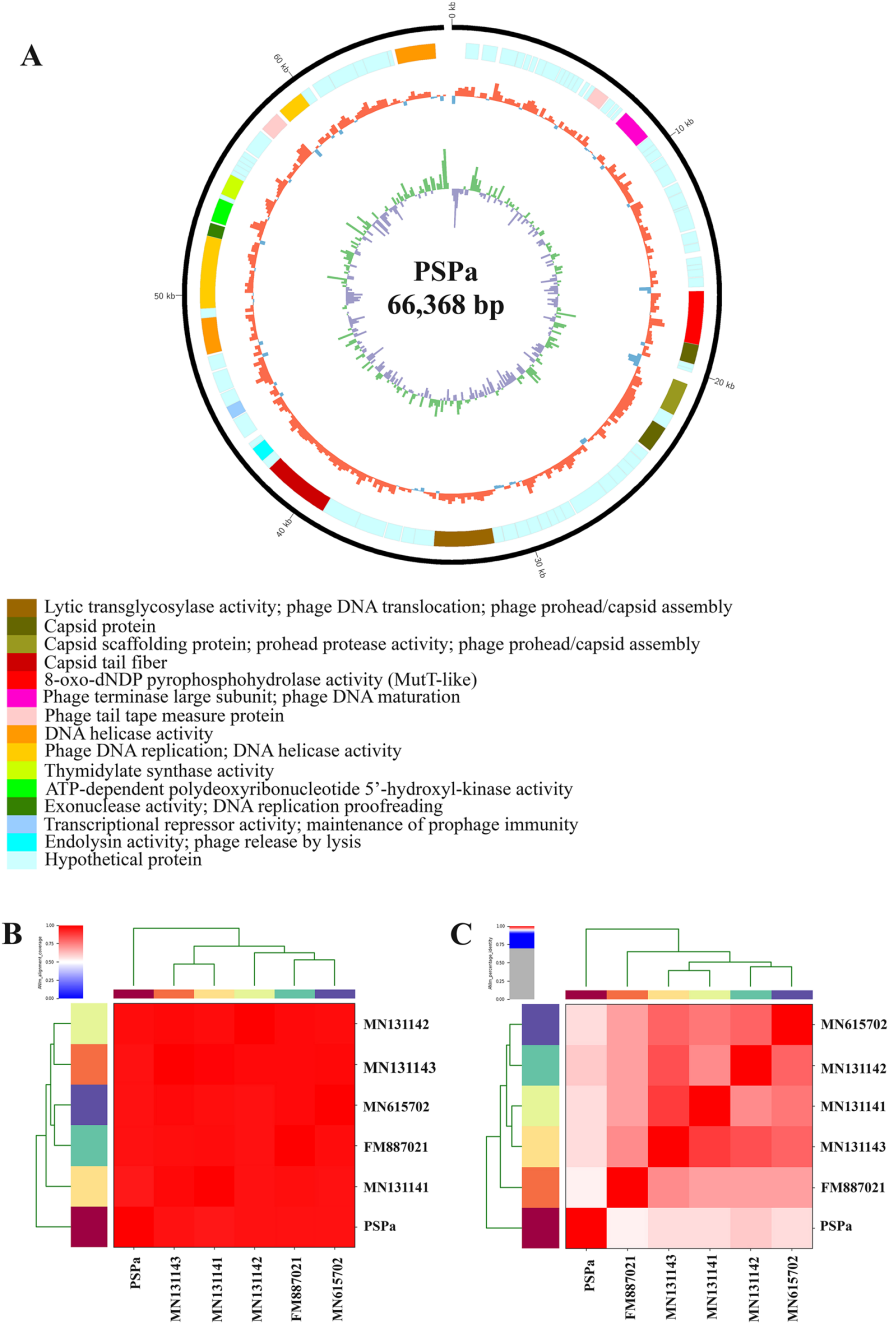
Genomic characterization of PSPa phage. (**A**) Genome organization map of PSPa; (**C**) alignment coverage of PSPa and (**B**) percentage identity of PSPa with *Pseudomonas* viruses PA11P1, PA8P1, and PaP1_EPu-2019, *Pseudomonas* phage SN and *Pseudomonas* phage vB_PaeM_SMS29 (MN131143.1, MN131142.1 and MN131141.1, FM887021.1 and MN615702.1).

Phages (PSPa and APPa) DNA were extracted using standard PCI method and the whole genome of isolated DNAs were sequenced. Upon analysis, PSPa phage genome was identified to be in the length of 66,368 bp with GC content of 55.58%. BLAST analysis of PSPa genome revealed (Fig. 2A) to have genome similarity of 97% with *Pseudomonas* viruses PA11P1, PA8P1, and PaP1_EPu-2019 (MN131143.1, MN131142.1 and 96% with *Pseudomonas* phage SN and *Pseudomonas* phage vB_PaeM_SMS29 (FM887021.1 and MN615702.1) (Supplemental Table S3). While functionally annotating against various non-redundant databases, the genome was found to have 92 ORF starting at 339 bp and continued until 66,277 bp of the sequence, 32 of which were of known functions and 60 of which were of hypothetical functions (Supplemental Table S1). Important genes that play a critical role in the infectivity and functionality of the phage were found within the genome of PSPa.

**Figure 3:**
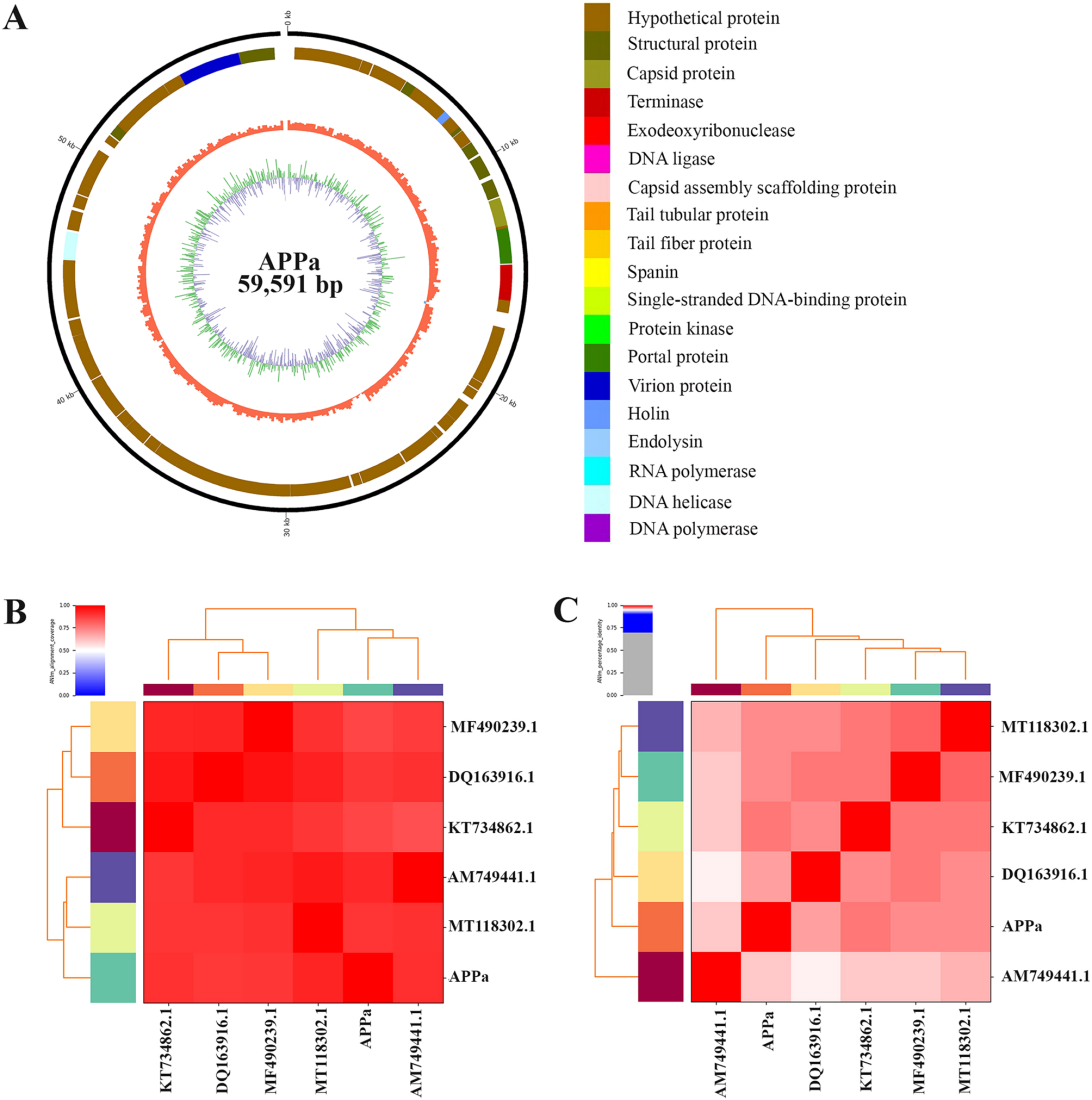
Genomic characterization of APPa phage. (**A**) Genome organization map of APPa created; (**C**) alignment coverage of APPa and (**B**) percentage identity of APPa with *Pseudomonas* phages vB_PaeS_S218, PAE1, Epa38, M6 and YuA (DQ163916.1, MF490239.1, KT734862.1, MT118302.1 and AM749441.1).

**Figure 4:**
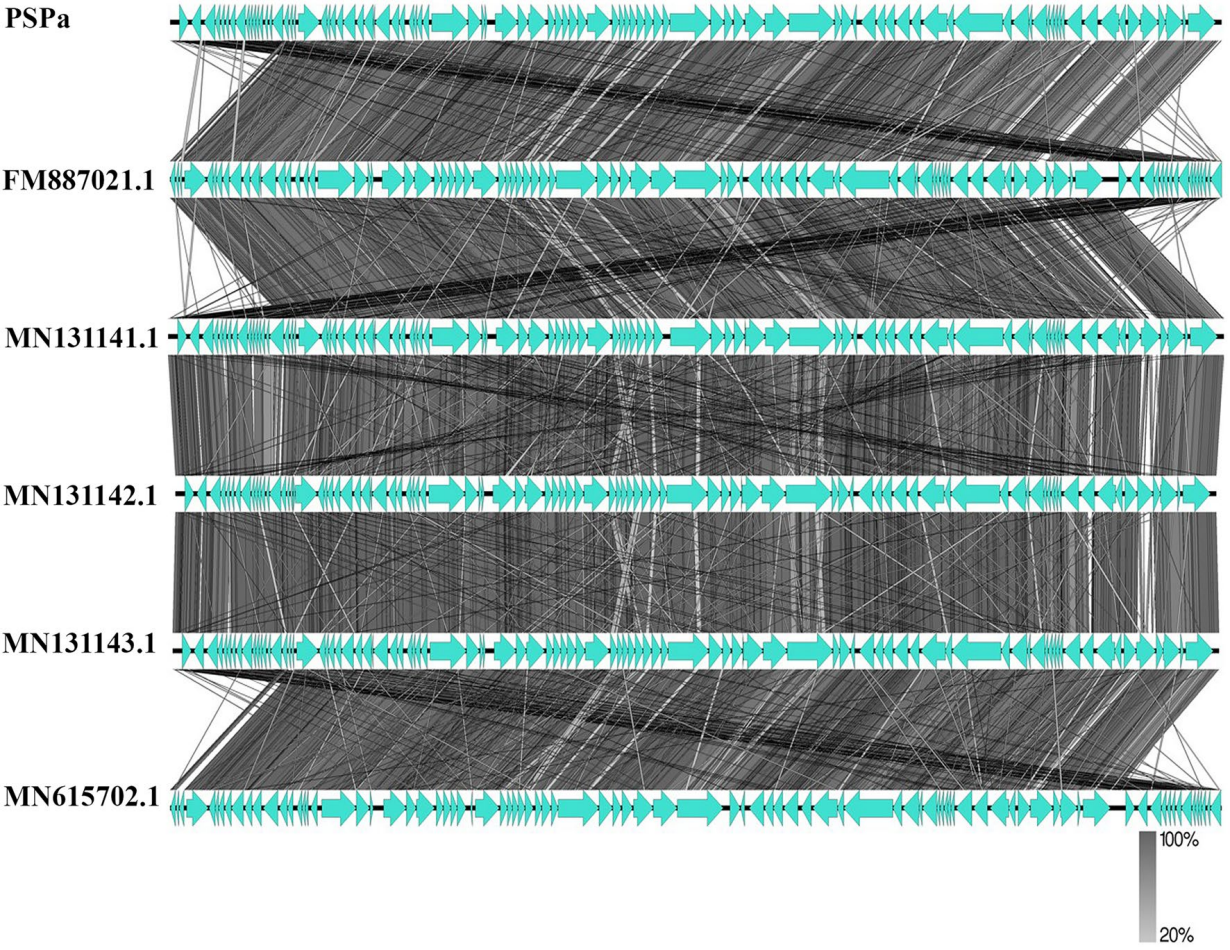
Comparative genome alignment of PSPa showing its evolutionary relationship with *Pseudomonas* viruses PA11P1, PA8P1, and PaP1_EPu-2019, *Pseudomonas* phage SN and *Pseudomonas* phage vB_PaeM_SMS29 (MN131143.1, MN131142.1 and MN131141.1, FM887021.1 and MN615702.1).

**Figure 5:**
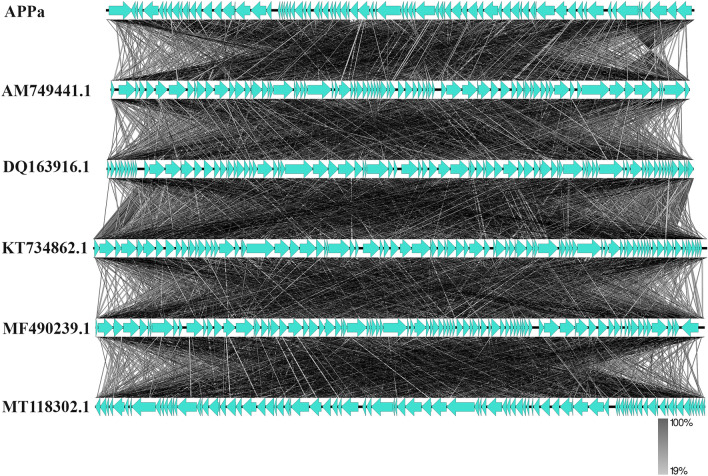
Comparative genome alignment of APPa with other *Pseudomonas* phages vB_PaeS_S218, PAE1, Epa38, M6 and YuA (DQ163916.1, MF490239.1, KT734862.1, MT118302.1 and AM749441.1).

Meanwhile, APPa phage genome was found to be in the length of 59,591 bp with GC content of 64%. Upon BLAST analysis, it was found that APPa shares 97% of homology with *Pseudomonas* phage Epa38 (MT118302.1) (Supplemental Table S3). Functional annotation of APPa genome revealed (Fig. 3A) the presence of 79 open-reading frames starting from 306 to 59318 bp responsible for coding 15 known and 64 hypothetical proteins (Supplemental Table S2). Phylogenetic analysis of phages PSPa and APPa revealed their evolutionary relationship with closely related phages of their respective families through percentage identity, shown in Figs. 2C and 3C (0–70% Grey, 70–90% Blue, 91–96% Pink, 97–100% Red) (Supplemental Table S5) and alignment coverage Figs. 2B and 3B (0–50% coverage Blue, 50% White, 75–100% Red) (Supplemental Table S4). Results of comparative genome organization analysis (Figs. 4 and 5), through Easyfig software clearly displayed the relationship between the genomes of identified phages and their related phages.

### In vitro therapeutic efficacy assessment

#### Susceptibility of *P. aeruginosa* planktonic cells

**Figure 6:**
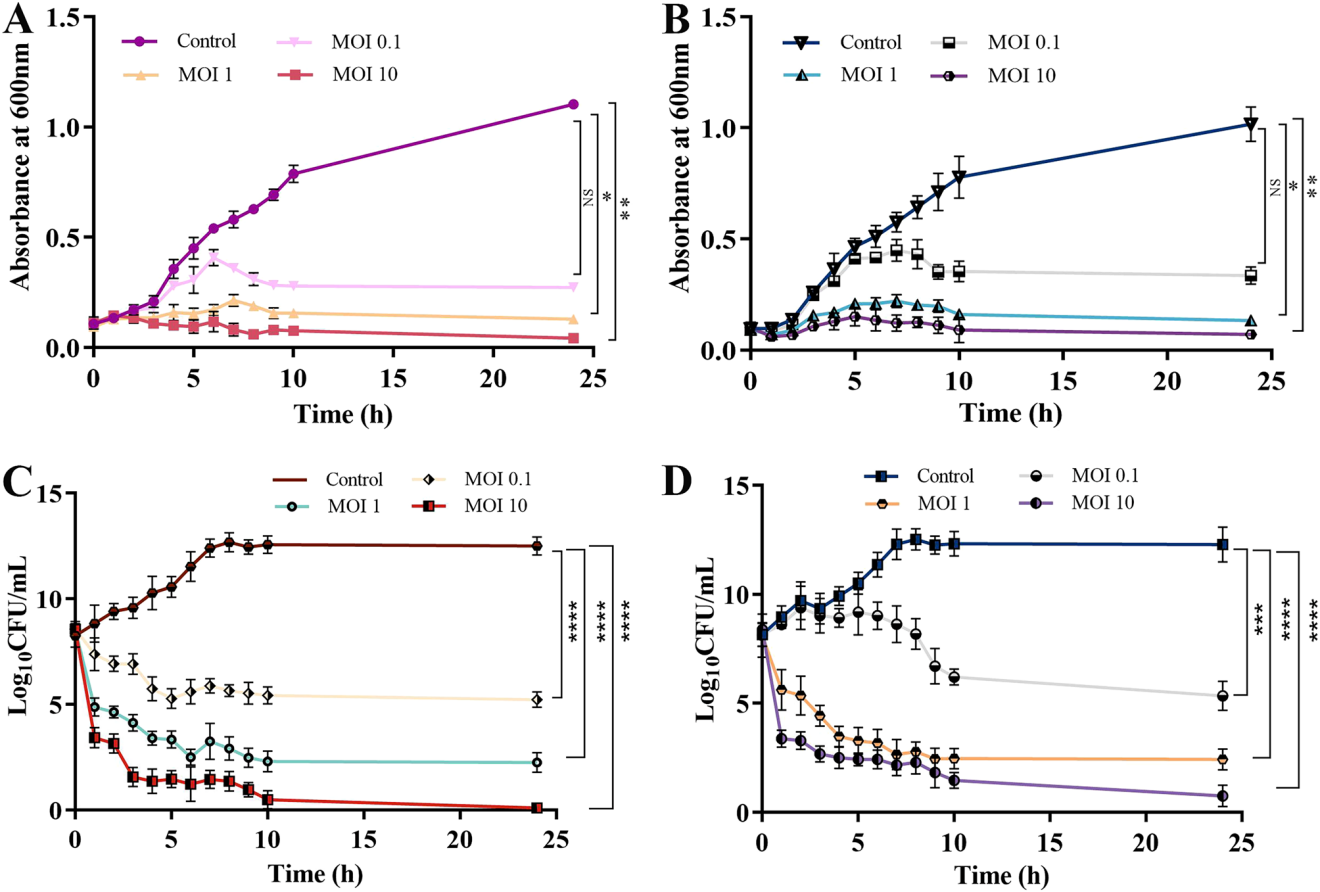
Therapeutic characterization of the two phages. (**A**) The activity of the phage PSPa and (**B**) APPa against planktonic cells of PAO1 (ATCC 15692) at OD_600_; (**C**) the CFU/mL of the phage PSPa and (**D**) APPa against planktonic cells of PAO1. The *, **, *** and **** denotes the significant values *P* < 0.05, *P* < 0.01,* P* < 0.001 and *P* < 0.0001, respectively.

The virulent activity of the phages PSPa and APPa was tested at various MOIs (0.1, 1, and 10) for 24 h in the presence of uninfected PAO1 as a control. The bacteria treated with phages at MOI 1 (*P* < 0.05) and 10 (*P* < 0.01) showed significant reduction in growth rate compared to the untreated control sample (Fig. 6A (PSPa) and B (APPa)). After 24 h both the phages depicted a maximum growth inhibition (~ 90%) at MOI 10. At MOI 10, 8.09 (PSPa), 7.38 (APPa) log CFU/mL reduction (*P* < 0.0001) was observed in the control and treated samples (Fig. 6C (PSPa) and D (APPa)).

#### Susceptibility of biofilms to phages PSPa and APPa

**Figure 7:**
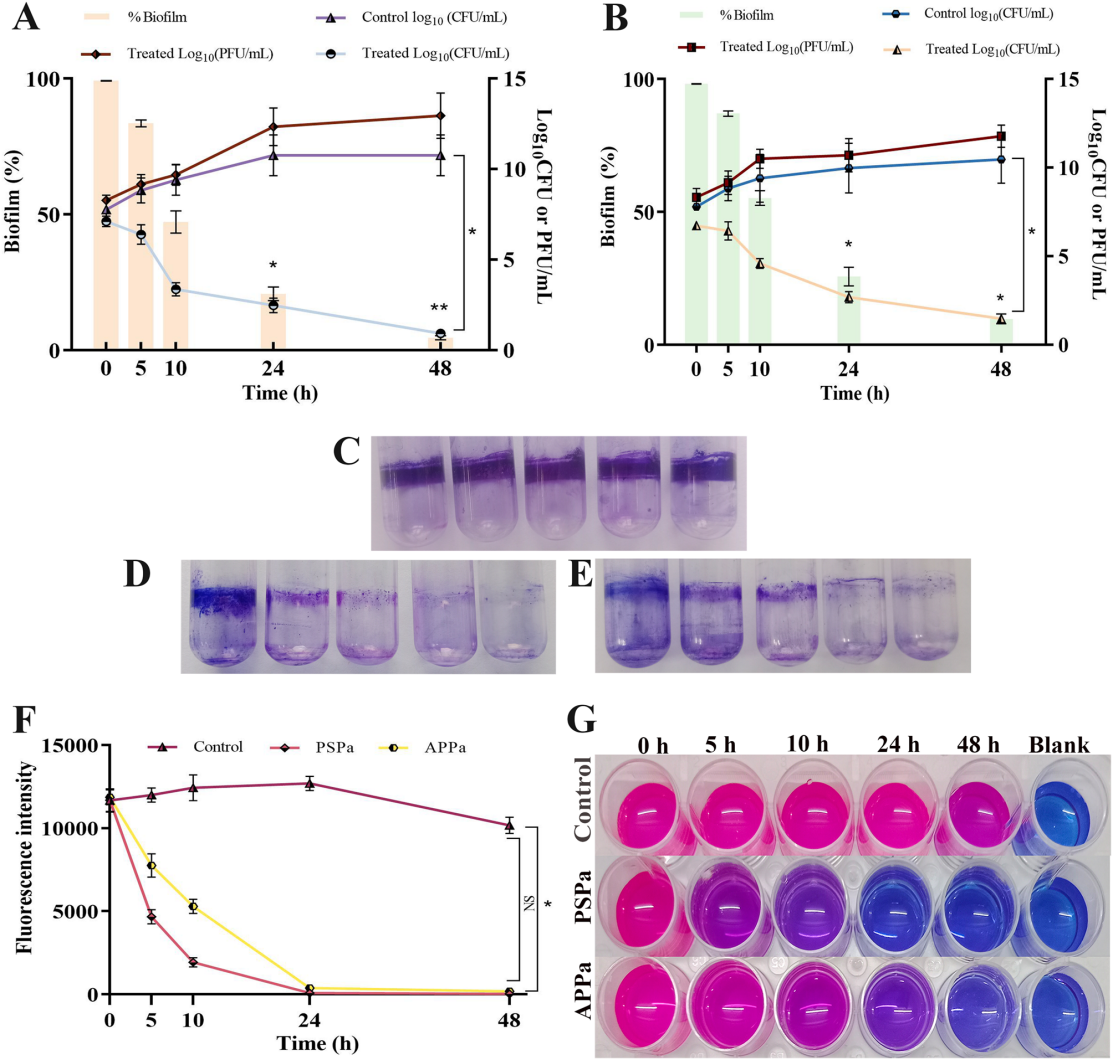
(**A**) Inhibitory potential of PSPa and (**B**) APPa on PAO1 (ATCC 15692) biofilms upon treatment over 24 h at 1.31 × 10^8^ PFU/mL; (**C** [Control], **D** [PSPa] and **E** [APPa]**)** Staining of biofilms with 1% crystal violet and total biofilm biomass quantification at OD570; (**F** and **G**) displays reduction in viability in PSPa and APPa phage treated biofilm cells of PAO1. The *, **, *** and **** denotes the significant values *P* < 0.05, *P* < 0.01,* P* < 0.001 and *P* < 0.0001, respectively. Error bars indicate the standard deviations. Each experiment was performed in triplicate, and the bars indicate standard deviations.

Data on PAO1 biofilm susceptibility to phage revealed that both the phages effectively reduced sessile cell density at the given PFU within 10 h (Fig. 7A–E). The most effectual biofilm reduction i.e., ~ 99.2% (PSPa) and ~ 98.1% (APPa) (*P* < 0.05) was observed at 48 h.

To further substantiate the above results, the cells were plated on LB agar and CFU was calculated (Fig. 7A and B). The number of bacterial cells were significantly decreased (*P* < 0.05) by ~ 5-fold log in the experiments involving both phages. In addition, the double-layer method enumerated phage growth and PFU was calculated. The number of phages present in the sample was increased up to ~ 5 & 4-fold log in the case of both PSPa and APPa phages, respectively. After 48 h, no reoccurrence of biofilm was observed. This result revealed that phages exhibited significant activity at MOI 10 (*P* < 0.05) against sessile cells of *P. aeruginosa* residing in biofilms.

#### Alamar blue assay

In the control samples, biofilm cells retained pink colour up to 48 h, indicating the presence of metabolically active cells (Fig. 7F and G). In case of phage-treated biofilm cells, the intensity of the pink colour reduced drastically with increasing time and changed to blue colour, signifying the phage-meditated lysis of metabolically active cells. Until 10 h, the pink colour was retained in PSPa-treated sample. Then, after 24 h until 48 h, colour change from pink to blue was observed. In addition, APPa phage-treated sample, the pink colour was retained up to 24 h, and even after 48 h of phage treatment, no pink colour was observed, clearly suggesting that almost 99% of the sessile cells were killed by phage’s action.

#### Microscopic analysis

**Figure 8:**
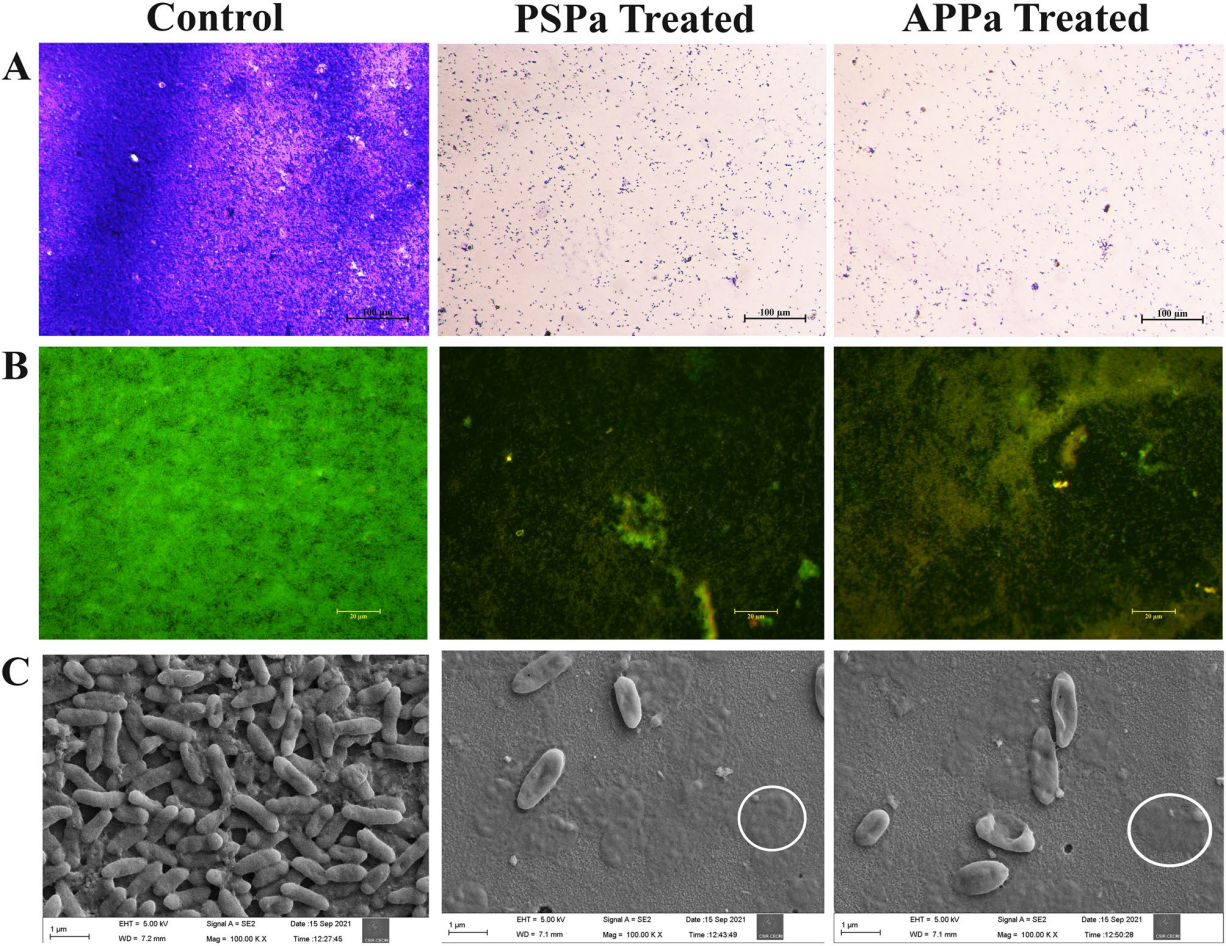
(**A**) Microscopic observations of infectivity of phages PSPa and APPa on *P. aeruginosa* (ATCC 15692) biofilms through light microscopy (the scale bar = 100 µm), (**B**) fluorescent microscopy using EtBr and AO (the scale bar = 20 µm) and (**C**) FE-SEM micrograph showing punctured and damaged cells of *P. aeruginosa* upon treatment with PSPa and APPa; circled regions highlights the completely killed *P. aeruginosa* cells (the scale bar = 1 µm).

The effect of PSPa and APPa phages (1.31 × 10^8^ PFU/mL) on PAO1 biofilm cells were microscopically examined using light, fluorescence and field emission scanning electron microscopies (Fig. 8).

The light micrographs of control sample depicted the presence densely packed multilayered biofilm cells enmeshed in extracellular polymeric substances**.** On the other hand, the micrographs of phage treated samples displayed scattered, disintegrated structure of cell arrangement (Fig. 8A).

In order to qualitatively examine the phage mediated lysis of biofilm cells, live dead staining (Fig. 8B) was performed deploying fluorescence microscope, wherein the cells were stained with two fluorescent dyes AO and EtBr. Resulting fluorescence micrographs obviously demonstrated the phenomenal lytic efficacy of phages on sessile cells of PAO1, as the untreated control samples portrayed green fluoresced cells i.e., live cells and samples treated with phages displayed intense red fluorescence, a typical indication of dead cells.

Further, to substantiate the phage’s lytic efficacy over sessile cells of PAO1, FE-SEM analysis was done. The presence of punctured and wrinkled sessile cells in phage-treated sample (Fig. 8C) reinforced the remarkable lytic ability of phages against biofilm cells of PAO1**.** In addition, the reduced production of EPS and the presence of disorganized cellular structure were also apparent in PSPa and APPa treated groups than that of the control group.

#### Effective eradication of persister cells of PAO1 by phages

**Figure 9:**
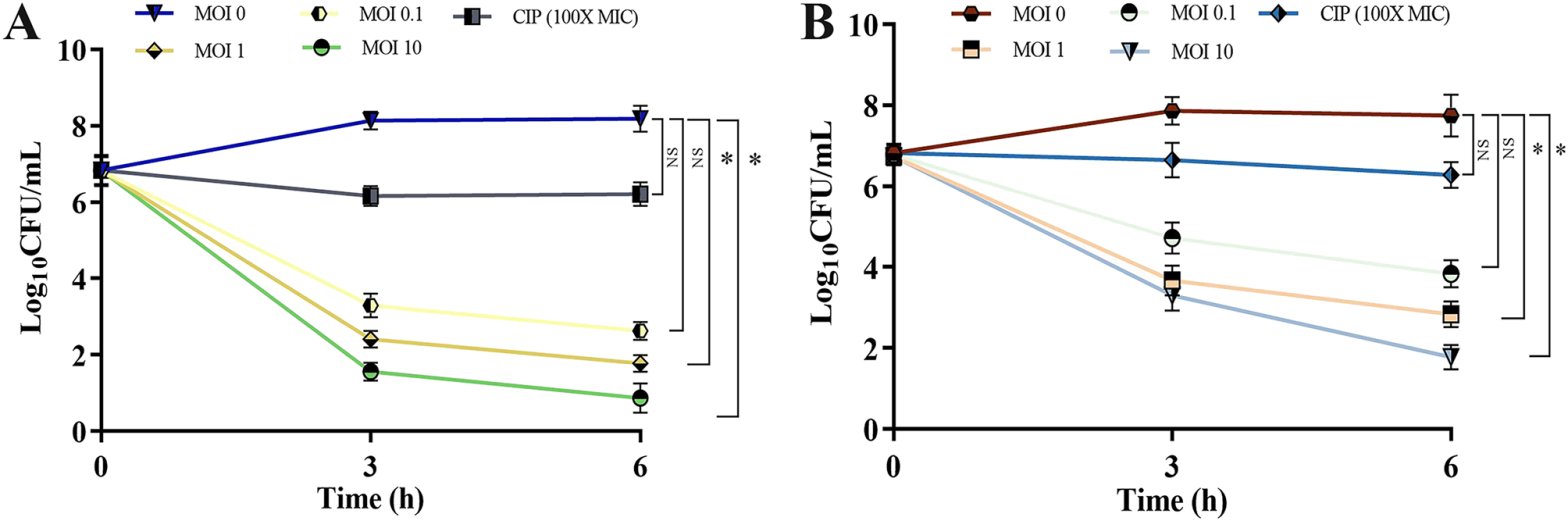
(**A**) PSPa and (**B**) APPa phages showing strong bactericidal activity against PAO1 (ATCC 15692) persisters induced by CIP at 100 × MIC. All the experiments were conducted in triplicate; error bars represent means ± SD and the *, **, *** and **** denotes the significant values *P* < 0.05, *P* < 0.01,* P* < 0.001 and *P* < 0.0001, respectively.

Initially, the susceptibility of biofilm-embedded PAO1 cells towards CIP antibiotic was tested and MIC of CIP was determined. Upon 24 h of treatment with 100X MIC of CIP, planktonic cells were vulnerable to the tested antibiotic, but sessile cells, which were deeply embedded in the biofilm was resistant; which indicated their persistent nature. The remnant persister cells that escaped 100× MIC CIP treatments were treated with phages (PSPa and APPa, individually) and CIP. Interestingly, eight-fold to six-fold reduction of persister cells were achieved upon treatment with PSPa and APPa phages (Fig. 9A and B), in the exposure duration of 6 h. whereas, only two-fold killing of biofilm-residing persister cells were achieved by CIP**.** CFU counting was used to assess cell viability following phage treatment. Even after prolonged incubation of the agar plates, there was no viable bacterial regeneration. Interestingly, there was no re-growth, even after 24 h when the treated cells from each condition were injected into fresh media. When these cultures were plated, no colonies were found. These findings evidently prove the ability of the phages to destroy PAO1 biofilm-residing persister cells.

### In vivo therapeutic characterization

#### Demonstration of in vivo therapeutic ability and expansion of survival of zebrafish infected with PAO1 by PSPa and APPa

**Figure 10:**
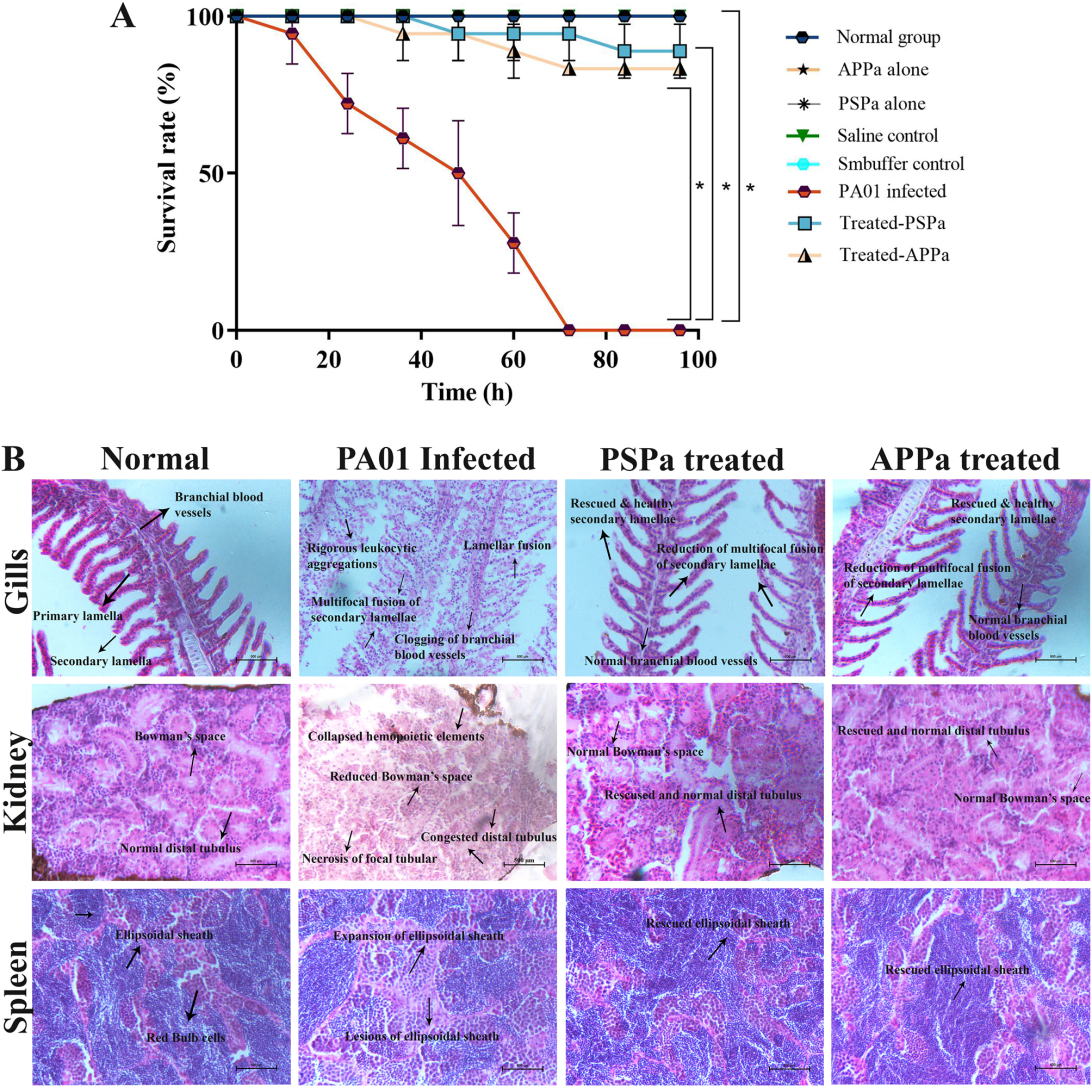
In vivo characterization of the two phages. (**A**) Phages PSPa and APPa displaying non-toxic nature to zebrafish and (**B**) improve the lifespan of post*-*infected zebrafish. (**C**) PSPa and APPa reduce tissue damage arose in organs such as gills, kidney and spleen of PAO1 ATCC (15692) infected zebrafish. The *, **, *** and **** denotes the significant values *P* < 0.05, *P* < 0.01, *P* < 0.001 and *P* < 0.0001, respectively.

The result of the toxicity study with zebrafish revealed that the group treated with isolated phages (PSPa and APPa) exhibited 100% survival at MOI 10 (Fig. 10A). The obtained result indicates that MOI 10 of PSPa and APPa phages did not show any toxic effects on the zebrafish, revealing their non-toxic nature. Therefore, PSPa and APPa at MOI 10 were selected for subsequent studies.

Survival assay was performed to test the in vivo anti-infective potential of isolated phages against *P. aeruginosa* PAO1. The assay was done with 12 h post-infected fish. The result revealed that the fish infected with *P. aeruginosa* PAO1 without any treatment resulted in complete mortality within 72 h (Fig. 10A). Whereas the infected fish treated with PSPa and APPa (at MOI 10) showed significant increase (*P* < 0.05) in the survival rate to the level of 88.89% and 83.33%, respectively, which suggests the therapeutic efficiency of isolated phages against PAO1 infection.

#### Histopathology analysis of PAO1 infected zebrafish treated with PSPa and APPa

Histopathology analysis, to observe damages caused due to systemic infection of PAO1 strain of *P. aeruginosa*, was performed. In Fig. 10B, the histopathology of the gill illustrates that PAO1 caused significant pathological alterations such as bulging of the primary gill, lamellar fusion, and a multifocal fusion of secondary lamella, congestion of branchial blood vessels and necrosis of erythrocytes in the gills in infected fish. Meanwhile, PSPa and APPa treated fish have natural and rescued gill composition similar to untreated control. Further, the kidney of infected fish had histological alterations such as reduced Bowman’s space, collapsed proximal or distal tubules and necrosis of hematopoietic tissues. Whereas, treated groups exhibited normal and healthy proximal or distal tubules and Bowman's space. The spleen of normal control showed regular construction of red pulp cells and compacted ellipsoidal cells. In *P. aeruginosa* PAO1 infected fish, rational splenomegaly was observed with a slight expansion of ellipsoid sheaths. PSPa and APPa treated fish were rescued from pathological lesions of ellipsoid cells and the architecture of red pulp cells. Overall, the histopathology results strongly suggest that the treatment of PSPa and APPa successfully rescued the infected animals from the pathognomonic impact of *P. aeruginosa* PAO1 infection.

## Discussion

In clinical settings, controlling *P. aeruginosa* mediated biofilm infections is imperative, as it imperils the health of immunocompromised patients by being the primary source of infections after surgery. In addition, its inherent ability to incite complications in illness such as pneumonitis and cystic fibrosis are huge public health menace that needs to be addressed worldwide. Besides, its resistance manifestation towards conventional antibiotics is yet another global crisis that requires urgency in the development of antibiotic substitutes^35^. In this milieu, phage therapy has been considered as one of the viable alternatives to antibiotics to address the issue of recurrent infections associated with *P. aeruginosa*.

In light of this, the current study focuses on the isolation of the phages PSPa and APPa, which are then characterized in terms of their phenotypic, genotypic and therapeutic properties. Both PSPa and APPa displayed clear, halo plaque morphology when challenged with *P. aeruginosa* PAO1. The structural interpretation using ICTV guidelines through TEM as well as phylogenetic analyses of the phages suggested PSPa and APPa to the family *Autographiviridae* and *Straboviridae*, to be the members of the class *Caudoviricetes*, confirming that they are tailed, dsDNA containing phages (Fig. 1B). PSPa shares structural similarities with previously characterized tailed phages such as PAtk1 and PAtk6^36^, and APPa shares morphological similarities with well-characterized structurally similar phages Bϕ-R656 and Bϕ-R1836^19^. Enhanced adsorption rate and one-step growth kinetics^37^, which are essential prerequisite for a phage to be used as a therapeutic, demonstrated that PSPa and APPa were highly active against *P. aeruginosa*. Interestingly, PSPa displayed a larger burst size than APPa, which could be attributed to PSPa’s rapid adsorption to *P. aeruginosa* than APPa (Fig. 1C and D). Notably, PSPa and APPa had depicted host specificity at the species level and lytic efficacy against 28 clinical MDR isolates of *P. aeruginosa*, as divulged through host-range analysis and EOP. Comparatively, phages PSPa and APPa had a larger burst size than the reported *Pseudomonas* phages such as C11 (11 PFU/infected cell)^38^ and JG024 (180 PFU/infected cell)^39^. These findings on PSPa and APPa have in turn signified their plausibility to be a viable antibiotic substitute of future in clinical settings.

Stability of phages under varying pH and temperature conditions, endorses their host-lytic behaviour including adsorption, infectivity, intracellular replication and multiplication^40^. In line to this, the thermostable and pH tolerant nature of both the phages under a range of temperature (4–65 °C) as well as pH^3–12^ evidenced their proficient bacterial lytic efficacy under adverse physiological conditions (Fig. 1E and F).

Genomic characterization of PSPa and APPa assisted in deepening our understanding on their physiological properties. It further facilitates to determine and validate their taxonomical identity and phylogenetic relationship with similar *Pseudomonas* phages of their respective family. Genomes of PSPa and APPa had percentage identity of 95 with the other reported *Pseudomonas* phages. The phages PSPa and APPa shared > 95% similarity with other related phages [*Pseudomonas* virus PA11P1, *Pseudomonas* phage SN, *Pseudomonas* virus PA8P1, *Pseudomonas* virus PaP1_EPu-2019 and *Pseudomonas* phage vB_PaeM_SMS29 (FM887021, MN131143, MN131142, MN131141 and MN615702) with PSPa; *Pseudomonas* phage vB_PaeS_S218,  *Pseudomonas* virus M6, *Pseudomonas* phage PAE1, *Pseudomonas* virus Yua and *Pseudomonas* phage Epa38 (MF490239.1, DQ163916.1, KT734862.1, AM749441.1 and MT118302.1) with APPa], which further confirms the host selectivity and phylogeny of the phages. The distribution of genes and comparison of genome organization for both of the phages PSPa and APPa with their selected relatives further confirmed their phylogenic identity as *Pseudomonas* phages (Figs. 2 and 3). The information on ORF annotation of the phage genome revealed the virulent nature of identified phages as they lack genes for lysogeny (Tables S1 and S2). Besides, it is noteworthy to observe that the genome of both PSPa and APPa were devoid of genes responsible for virulence as well as antibiotic resistance. Due to their inherent nature as lytic phages, the possibility of acquiring their host’s virulent genes through DNA recombination are close to none, which their supports their clinical safety to be used as therapeutics. Both PSPa and APPa harbored over 10 genes encoding structural proteins that are crucially responsible for structural framework of the phages. In addition, DNA helicase, which facilitates dsDNA unwinding during replication^41^, was found in both PSPa (ORF 64 and 85) and APPa (ORF 66). Despite its essential role in DNA replication, DNA helicase in turn assist in rapid and seamless multiplication of phages and maintain their virulent nature. Importantly, the two endonuclease genes (ORFs 49 and 82) that degrade foreign DNA were present in the PSPa genome, which limits the possibility of acquiring virulence or antibiotic resistance genes from the host bacteria.

Furthermore, PSPa genome harbored endolysin (ORF 92 in PSPa: identified as glycosyl hydrolase enzyme-family 19), a key gene responsible for progeny release through peptidoglycan degradation. Similarly, both the phages contained holin gene (ORF 69 in PSPa; ORF 10 in APPa), which is responsible for creating pores in the inner membrane to facilitate peptidoglycan access to the phage encoded endolysin^42^. Thus, it is envisaged from the present study that these enzymes could potentially be deployed as an enzybiotic of future. The analysis of genomic organization of these phages emphasizes their evolutionary relationship with the other reported phages (Fig. 4 and 5).

PSPa and APPa controlled the planktonic cell population of MDR *P. aeruginosa* with increasing MOI (Fig. 6). Phages have the ability to penetrate recalcitrant biofilm by eliminating biofilm residing sessile cells and collapsing biofilm integrity^43^. In par with this, PSPa and APPa effectively suppressed the growth of biofilm cells at 1.31 × 10^8^ PFU/mL (Fig. 7A–E), which was further substantiated through Alamar blue assay divulging the reduced metabolic status of phage treated mature biofilm cells (Fig. 7F and G). Administration of phages PSPa and APPa were able to significantly diminish PAO1 biofilm cells’ viability up to ~ 99.2% and ~ 98.1% respectively (Fig. 7A and B), which is comparatively superior to the earlier reported phage PEV20, which exhibited 74% of biofilm reduction in combination with CIP against clinical isolates of *P. aeruginosa*^44^.

Most importantly, PSPa and APPa were able to eradicate 24 h biofilm up to a significant level even in the minimal treatment period of 6 h, which reflects a comparably superior efficacy to that of the earlier reported phages MAG1 & MAG4 by Kwiatek et al.^45^ and phages vB_PaeM_SCUT-S1 & vB_PaeM_SCUT-S2 characterized by Guo et al.^46^ exhibited nearly 60% and 70% biofilm reduction, respectively. Notably, both PSPa and APPa also achieved higher biofilm inhibitory potency within 6 h, even in 48 h biofilms of *P. aeruginosa*. Further, the biofilm reduction ability of the identified phages was validated using light, fluorescence and FE-SEM microscopic techniques, providing insight into phage-mediated disruption, disintegration, and de-agglomeration of biofilm architecture (Fig. 8).

Persister cells in biofilms being a crucial player in recurrent infection by exuding high drug tolerance^47^, any agent having proficiency to kill these cells is a therapeutic mandate in the current multidrug resistance era. In the present study, PSPa and APPa phages greatly influenced the persister population of *P. aeruginosa* even at the lowest of their MOIs (Fig. 9). Due to the paucity of studies focusing on phage therapy against the persister population of *P. aeruginosa*, the current work finds an advantage in establishing PSPa and APPa phages as potential candidates for the treatment of persistent infection of MDR *P. aeruginosa*.

Zebrafish (*Danio rerio*) has been regarded as an ideal in vivo model for studying the efficacy of therapeutics in the treatment of various human diseases, owing to its high conservation with mammals^48^. Here, the in vivo efficacy evaluation of PSPa and APPa demonstrated an improved survivability of *P. aeruginosa* infected zebrafish by reducing bacterial abundance, which was further substantiated through histopathological examination, highlighting reduced tissue damage upon recovery from *P. aeruginosa* infection (Fig. 10). The in vivo data of the present study is in total agreement with a previous report on cystic fibrosis zebrafish embryo model infected with *P. aeruginosa*, wherein treatment with a cocktail of four phages demonstrated significant reduction in lethality from 83 to 52%^49^. Besides, several in vivo efficacy evaluation studies on phages has been published using model organisms such *Galleria mellonella* and mice; however, reports on zebrafish as model against *P. aeruginosa* is really scanty^19,50^.

## Conclusion

The present study demonstrates phenotypic, genotypic and therapeutic (in vitro and in vivo) propensities of two newly isolated phages PSPa and APPa specific to *P. aeruginosa* PAO1 and its MDR clinical isolates. Both the phages were identified to exhibit inhibitory potential over sessile and persister cells of *P. aeruginosa*. Through this study, we propose that the phages PSPa and APPa could be exploited as candidates of viable substitute to the existing means of antimicrobial therapy to treat *P. aeruginosa* related infections in near future.

### Supplementary Information

Supplementary Figure 1.

Supplementary Figure 2.

Supplementary Figure 3.

Supplementary Tables.

## Data Availability

The sequencing data used in this study are given as Supplementary file (Supplementary Table.1.xls). All other datasets generated and/or analysed during the current study are available from the corresponding author on reasonable request.
