# Supplementary figures and images for: Two novel phages PSPa and APPa inhibit planktonic, sessile and persister populations of Pseudomonas aeruginosa, and mitigate its virulence in Zebrafish model
Source: Sci Rep. 2023 Nov 3;13:19033. doi: 10.1038/s41598-023-45313-x (PMC10624879; doi:10.1038/s41598-023-45313-x)

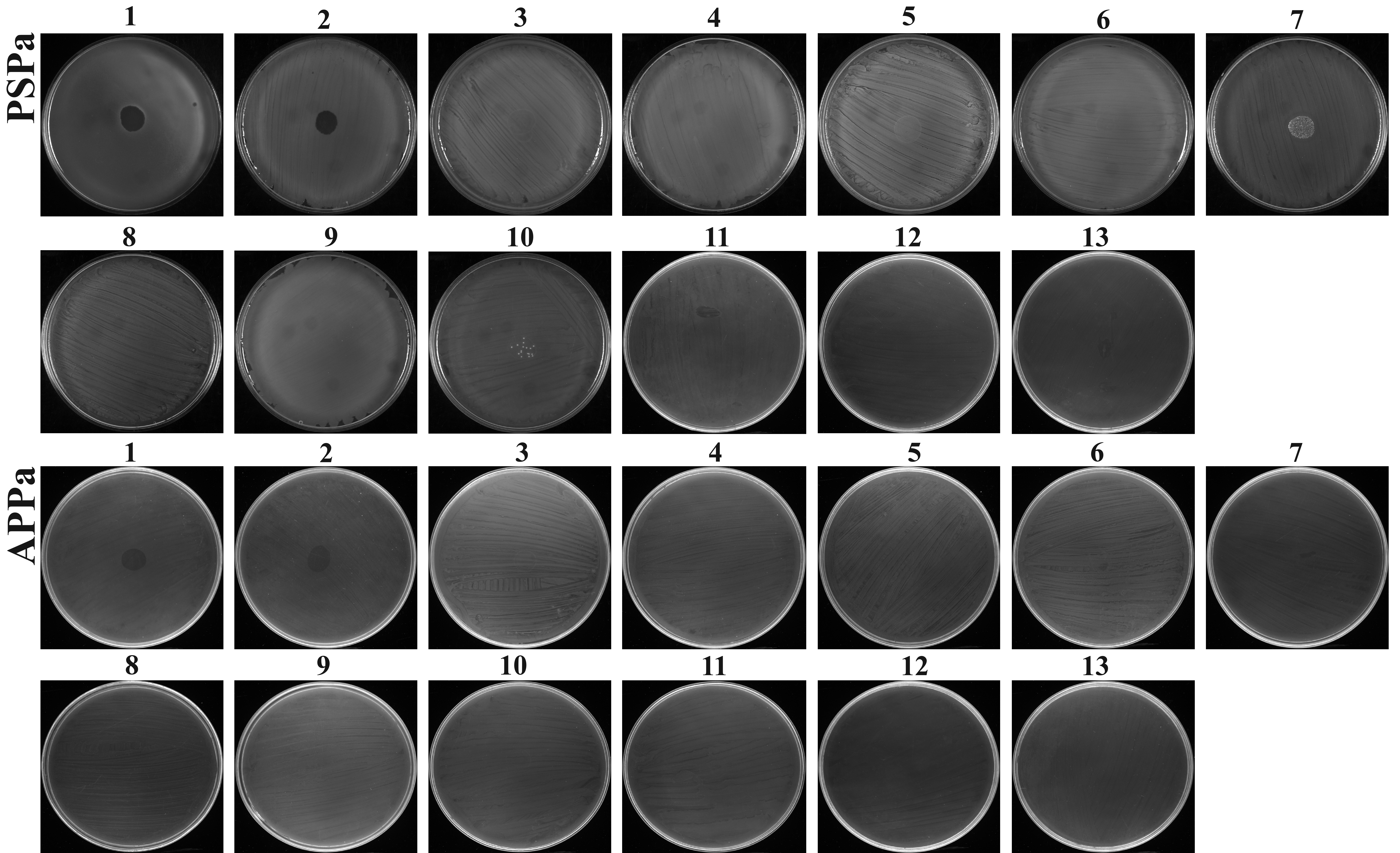

Supplement: Supplementary file 1 — Supplementary Figure 1. [file 41598_2023_45313_MOESM1_ESM.jpg]

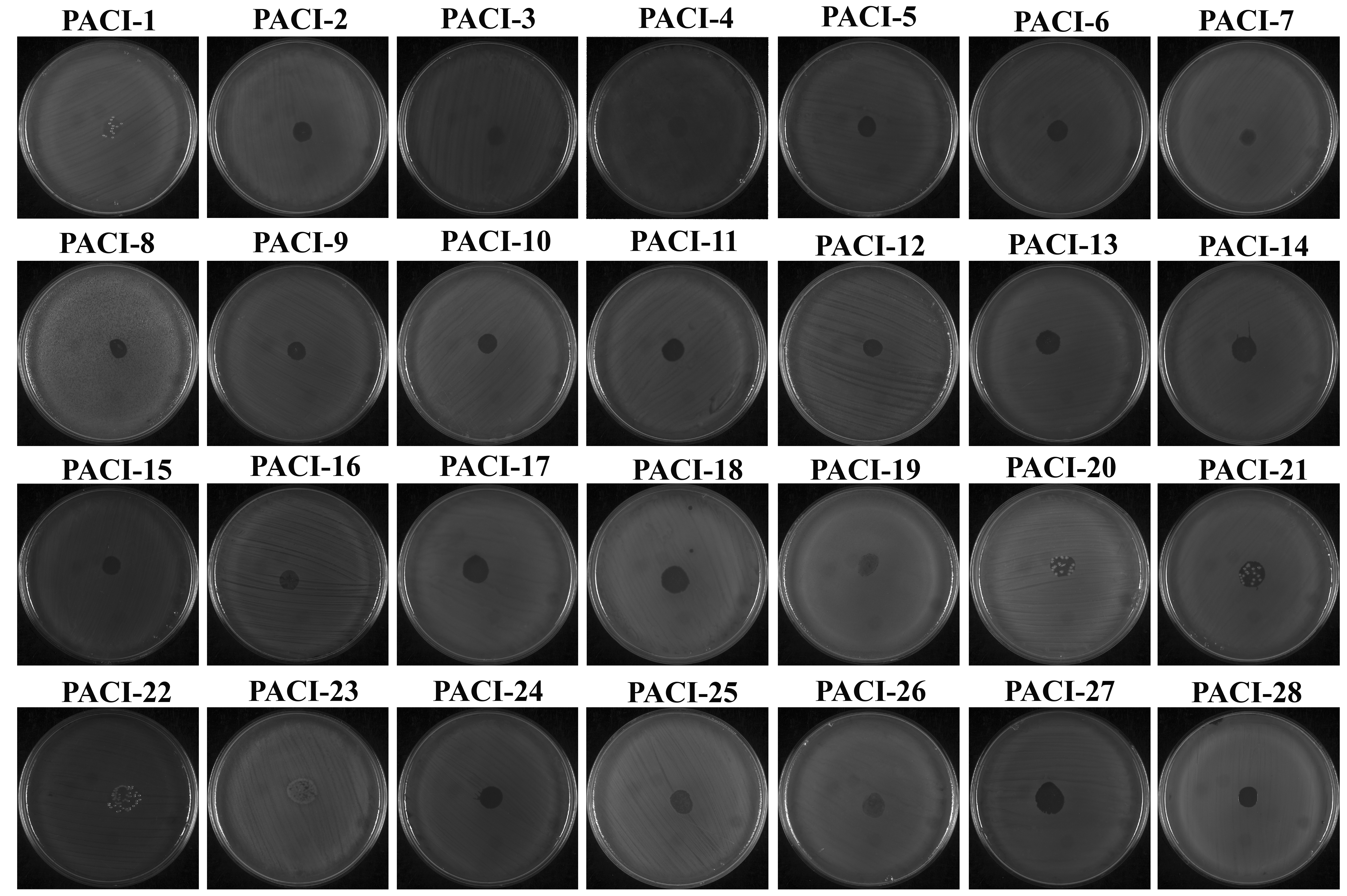

Supplement: Supplementary file 2 — Supplementary Figure 2. [file 41598_2023_45313_MOESM2_ESM.tif]

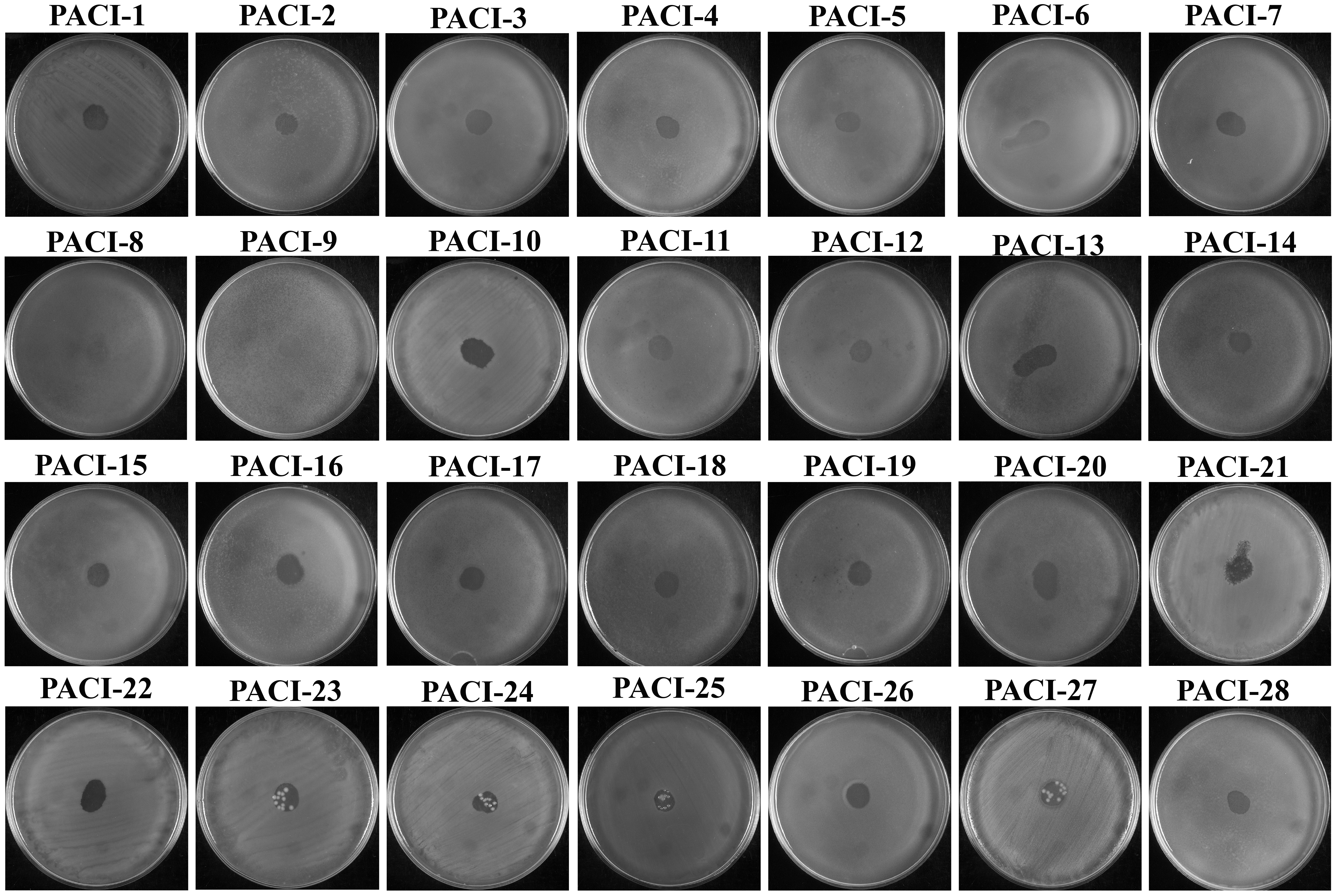

Supplement: Supplementary file 3 — Supplementary Figure 3. [file 41598_2023_45313_MOESM3_ESM.jpg]
